# Prevalence and Antimicrobial Resistance of *Escherichia coli* Isolates from Chicken Meat in Romania

**DOI:** 10.3390/ani13223488

**Published:** 2023-11-12

**Authors:** Dariana Olivia Brătfelan, Alexandra Tabaran, Liora Colobatiu, Romolica Mihaiu, Marian Mihaiu

**Affiliations:** 1 Department of Animal Breeding and Food Safety, Faculty of Veterinary Medicine, University of Agricultural Sciences and Veterinary Medicine, Manastur Street No. 3/5, 400372 Cluj-Napoca, Romania; dariana-olivia.bratfelan@usamvcluj.ro (D.O.B.); alexandra.lapusan@usamvcluj.ro (A.T.); marian.mihaiu@usamvcluj.ro (M.M.); 2 Department of Medical Devices, Faculty of Pharmacy, Iuliu Hatieganu University of Medicine and Pharmacy, Victor Babes Street No. 8, 400012 Cluj-Napoca, Romania; 3 Department of Management, Faculty of Economic Sciences and Business Administration, Babes Bolyai University, Mihail Kogalniceanu Street No.1, 400084 Cluj-Napoca, Romania; romolica.mihaiu@econ.ubbcluj.ro

**Keywords:** *Escherichia coli*, antimicrobial resistance, resistance patterns, food safety, chicken meat

## Abstract

**Simple Summary:**

In Romania, the consumption rate of chicken meat is high, while the broiler industry is a rapidly growing sector in the country. Chicken meat is often contaminated with various pathogenic bacteria, including *Escherichia coli*, which remains one of the most frequent causes of common bacterial infections in both animals and humans. The aim of the current study was to evaluate the prevalence of *Escherichia coli* in chicken meat samples, as well as to analyze the antimicrobial susceptibility of these isolates. An overall prevalence of 30% has been determined in our study, which seems to be lower compared to prevalence rates previously reported. However, the *Escherichia coli* isolates recovered in the current study showed substantial resistance to multiple antibiotic classes, including fluoroquinolones and third generation cephalosporins, which is concerning. The evaluation of the prevalence and antimicrobial resistance of *Escherichia coli* is highly important, both for food safety reasons, as well as for analyzing its public health impact and the spread of antimicrobial-resistant bacteria to humans.

**Abstract:**

The current study was conducted in order to analyze the prevalence of *Escherichia coli (E. coli)* in samples of chicken meat (100 chicken meat samples), as well as to evaluate the antimicrobial susceptibility of these isolates. A total of 30 samples were positive for *E. coli* among the collected chicken samples. Most isolates proved to be highly resistant to tetracycline (80%), ampicillin (80%), sulfamethoxazole (73.33%), chloramphenicol (70%) and nalidixic acid (60%). Strong resistance to ciprofloxacin (56.66%), trimethoprim (50%), cefotaxime (46.66%), ceftazidime (43.33%) and gentamicin (40%) was also observed. Notably, one *E. coli* strain also proved to be resistant to colistin. The antimicrobial resistance determinants detected among the *E. coli* isolates recovered in our study were consistent with their resistance phenotypes. Most of the isolates harbored the *tetA* (53.33%), *tetB* (46.66%), *blaTEM* (36.66%) and *sul1* (26.66%) genes, but also *aadA1* (23.33%), *blaCTX* (16.66%), *blaOXA* (16.66%), *qnrA* (16.66%) and *aac* (10%). In conclusion, to the best of our knowledge, this is among the first studies analyzing the prevalence and antimicrobial resistance of *E. coli* strains isolated from chicken meat in Romania and probably the first study reporting colistin resistance in *E. coli* isolates recovered from food sources in our country.

## 1. Introduction

*Escherichia coli* (*E. coli)* is a gram-negative bacterium, facultatively anaerobic and a member of the *Enterobacteriaceae* family [1,2]. It is naturally detected in the commensal flora of warm-blooded animals, as well as humans. Even though most of the isolates are commensal ones, *E. coli* still remains one of the most frequent causes of common bacterial infections in both animals and humans [1,3,4].

It is also considered to be a complex and versatile species, having diversified into pathotypes of zoonotic intestinal pathogenic *E. coli* (IPEC) and extraintestinal pathogenic *E. coli* (ExPEC), pathogenic strains causing various intestinal and extraintestinal infections in a wide range of hosts [5,6].

According to the World Health Organization (WHO), antimicrobial resistance (AMR) represents a major public health concern and one of the top ten global health problems faced by humanity today [7,8]. Infections caused by antimicrobial-resistant bacteria are associated with significant health complications worldwide, including ineffectiveness and failure of currently available treatments, prolonged hospitalization and mortality [8,9].

The spread of AMR is considered to be the most divisive issue when it comes to the health of humans, animals and ecosystems in the twenty-first century. Such spread has also emerged as a significant barrier to economic development [10,11].

One of the main factors implied in the emergence and spread of antimicrobial-resistant bacteria is related to the misuse or unregulated use of antimicrobials in veterinary and clinical settings [12]. In livestock, antimicrobials are administered in order to improve the health and production of animals, including poultry [13,14].

Due to its ubiquitous nature, *E. coli* is commonly used as an indicator bacteria of fecal contamination, as well as a model microorganism or a sentinel microorganism for detecting AMR, as well as for AMR surveillance, being abundant in a wide range of hosts and also able to acquire resistance easily [15,16,17]. The bacteria has developed different mechanisms of resistance against important antimicrobials, mostly due to its ability to transfer genes located on mobile genetic elements and genomic islands through horizontal gene transfer [17,18,19].

One of the main resistance mechanisms of Gram-negative bacteria such as *E. coli*, implies the production of various hydrolytically active beta-lactamases. The enzymatic hydrolysis profile, as well as host range are changing constantly, from chromosome-mediated, to plasmid-mediated AmpC beta lactamases [20,21].

AMR *E. coli* are broadly distributed in Europe and have been found in humans, food-producing animals or food sources, including poultry and poultry products [4]. Food may act as a vector for the transfer of antimicrobial-resistant bacteria and AMR genes to humans [22].

High resistance rates to clinically important antimicrobials such as third-generation cephalosporins, fluoroquinolones or colistin have been reported among isolates recovered from food-producing animals (frequently from broilers), as well as from chicken and turkey meat [23,24,25]. The emergence of such resistance, especially in isolates recovered from meat is worrisome and requires close monitoring, as meat represents a significant proportion of the human diet, with further increase in consumption being estimated in the near future [26,27].

Notably, colistin resistance has attracted much attention lately, even becoming a public health issue [28]. Colistin belongs to the family of polymyxins. Its use in veterinary medicine has been abandoned for a while, but it has been recently introduced in poultry and pig farming in order to prevent infections caused by Gram-negative bacteria [28]. In human medicine, colistin is currently used as a last resort antibiotic in the treatment of problematic infections caused by multidrug resistant Gram-negative bacteria, including in the treatment of isolates which are resistant to carbapenems. Over recent years, a gradual increase in colistin resistance has been noticed, which undermines its efficacy. Also, greater attention has been drawn to the mechanisms of acquisition of colistin resistance in various pathogens, including *E. coli* [29,30].

In Romania, the consumption rate of chicken meat is high, while the broiler industry is a rapidly growing sector in the country. Even though a few studies have shown that food-producing animals in Romania may represent a potential reservoir of AMR microorganisms, including *E. coli*, data regarding the prevalence and AMR of *E. coli* isolates recovered from chicken meat is limited [31,32,33].

Therefore, the aim of the current study was to analyze the prevalence of *E. coli* in samples of chicken meat, as well as to evaluate the antimicrobial susceptibility of these isolates. Moreover, the presence of several antimicrobial-resistance genes has also been detected.

## 2. Materials and Methods

### 2.1. Sampling

A total of 100 samples from chicken meat were included in the study. The samples were aseptically collected during September 2022 to June 2023 from three different slaughtering units located in the center of Romania, these representing the most important poultry slaughtering units in the region. All samples were transported to the laboratory within 3 h after collection.

### 2.2. E. coli Isolation and Identification

After proper homogenization, all samples were transferred into separate tubes containing Luria nutrient broth (LB) and afterwards cultured at 37 °C, according to the steps mentioned in the ISO 16654:2001 protocol [34]. Briefly, all samples were inoculated into MacConkey agar plates (Merck, Darmstadt, Germany), followed by overnight incubation at 37 °C. Typical *E. coli* isolates were selected from each sample for further analysis. Vitek^®^ 2 GN cards for identifying a broad range of Gram-negative *Enterobacteriaceae* (intended for use with the Vitek 2 system) (bioMérieux, Marcy l’Etoile, France) were further used for the biochemical confirmation of the strains.

### 2.3. Antimicrobial Susceptibility Testing

The minimum inhibitory concentration (MIC) of various antimicrobials was determined by the broth-dilution method, using an automated system (Sensititre, Trek Diagnostic Systems, Cleveland, OH, USA), as previously described [2,12]. The following 18 antimicrobial agents from 9 different classes were included in the susceptibility testing panel: nalidixic acid (NA), amikacin (AK), gentamicin (GEN), ampicillin (AMP), cefotaxime (CTX), ceftazidime (CAZ), cefepime (FEP), cefoxitin (FOX), ciprofloxacin (CIP), chloramphenicol (CHL), colistin (CST), imipenem (IPM), meropenem (MEM), ertapenem (ETP), tetracycline (TET), trimethoprim (TMT), sulfamethoxazole (SMX) and azithromycin (AZM). The MICs were interpreted according to the interpretive criteria described by the Clinical Laboratory Standards Institute (CLSI, 2018) [35]. No CLSI resistance breakpoints are available for AZM, therefore, previously reported interpretive criteria were used instead [2]. The *E. coli* isolates which proved to be resistant to more than three antimicrobial classes were considered as multidrug resistant (MDR).

### 2.4. Bacterial Genomic DNA Extraction

The total genomic DNA was extracted following a protocol previously described by Mihaiu et al. [36]. In brief, 3 specific *E. coli* isolates were removed from the MacConkey agar plates and then resuspended into 150 μL Chelex solution (Sigma Aldrich, St. Louis, MO, USA). The samples were afterwards subjected to a high temperature protocol for cell membrane lysis (94 °C for 15 min and 56 °C for 10 min). A Nanodrop ND-1000 spectrophotometer analyzer (NanoDrop Technologies, Wilmington, DE, USA) was further used in order to assess the quality and quantity of the extracted DNA.

### 2.5. Detection of Antimicrobial Resistance Genes

A multiplex PCR was employed in order to investigate the presence of antimicrobial resistance genes, namely *blaSHV*, *blaCMY*, *blaTEM*, *blaCTX*, *blaOXA* (β-lactamase genes), *qnrA* (quinolones), *aadA1* (aminoglycosides), *aac* (gentamicin), *sul1* (sulphonamides), *ere(A)* (erythromycin) and *tetA* and *tetB* (tetracyclines). The PCR protocol used was previously described by Chirilă et al. [37]. Briefly, the PCR reaction mix (25 μL) was comprised of: 1×PCR green Buffer, 2.5 mM MgCl2; 5 pmol of each primer, dNTPs each at 200 μM, 2.5U of TaqDNA polymerase (Promega), and 100 ng of genomic DNA. The analysis was performed under the following conditions: 94 °C for 3 min followed by 35 cycles of 94 °C for 30 s, 58 °C for 30 s, and 72 °C for 1 min, and a final extension step of 73 °C for 5 min. 10 μL of the amplified product were loaded into agarose gels (2%). The gels were then stained with EvaGreen (JenaBioscience, Jena, Germany) and electrophoresed (90 W) for 40 min. Visualization was performed under UV light with a Gel Doc XR+Imager (Bio-Rad, Hercules, CA, USA). Strains of MDR *E. coli* (O157:K88ac:H19, CAPM 5933) were used as positive controls. The primers used to detect the presence of the above-mentioned AMR genes have been previously reported [37].

## 3. Results

### 3.1. Prevalence of E. coli

Following the isolation protocol, a total of 30 *E. coli* isolates were recovered from the 100 analyzed samples (30/100; 30% prevalence).

### 3.2. Antimicrobial Susceptibility Testing

Almost all isolates recovered in the current study exhibited resistance phenotypes (96.66%).

**Figure 1:**
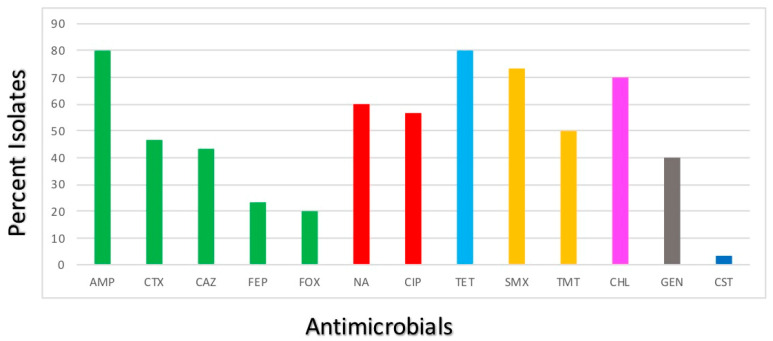
Antimicrobial resistance profiles of the *E. coli* isolates. Bars represent the percentage of the *E. coli* isolates showing resistance to 13 medically important antimicrobials. Green—β-lactams; Red—quinolones; Light blue—tetracyclines; Yellow—sulphonamides; Pink—amphenicols; Grey—aminoglycosides; Dark blue—polymyxins.

The susceptibility profiles of the *E. coli* isolates are presented in Figure 1.

The results indicate that all recovered *E. coli* were susceptible to AMK, IPM, MEM, ETP and AZT. Most isolates proved to be resistant to TET (24/30; 80%), AMP (24/30; 80%), SMX (22/30; 73.33%), CHL (21/30; 70%) and NA (18/30; 60%). Strong resistance to CIP (17/30; 56.66%), TMP (15/30; 50%), CTX (14/30; 46.66%), CAZ (13/30; 43.33%) and GEN (12/30; 40%) was also observed. The *E. coli* isolates showed low percentages of resistance to FEP (7/30; 23.33%), FOX (6/30; 20%) and CST (1/30; 3.33%). Moreover, more than 70% of the isolates proved to be multidrug resistant, showing resistance to at least three different classes of antibiotics.

### 3.3. Detection of Antimicrobial Resistance Genes

Consistent with their resistance phenotypes, antimicrobial resistance determinants detected among the *E. coli* isolates included *tetA* (16/30; 53.33%), *tetB* (14/100; 46.66%), *blaTEM* (11/30; 36.66%), *sul1* (8/100; 26.66%), *aadA1* (7/30; 23.33%), *blaCTX* (5/30; 16.66%), *blaOXA* (5/30; 16.66%), *qnrA* (5/30; 16.66%) and *aac* (3/30; 10%) (Table 1).

## 4. Discussion

Poultry meat represents an important component of our diet due to its nutritional value, while poultry meat production, as well as consumption, is rapidly growing worldwide. Unfortunately, poultry meat can be contaminated with various pathogenic bacteria which can be transmitted to humans, causing foodborne infections [26,27,38]. The presence of pathogenic microorganisms in food products is considered to be a worldwide public health problem, especially taking into consideration the potential of food products such as poultry, meat and dairy to transfer AMR bacteria, as well as AMR resistance to humans [6].

Overall, a total of 30 (30%) *E. coli* isolates were recovered from the 100 investigated samples. Different studies have analyzed the prevalence and distribution of *E. coli* in different countries of the world, including Brazil, Qatar, the United States, China, the United Arab Emirates, Canada, Japan, Egypt, India, Turkey, Italy or Belgium [1,6,8,12,27,39,40,41,42,43]. Many of these isolates were recovered from meat (especially chicken and turkey, but also duck, beef and pork).

The prevalence of *E. coli* identified in our study appears to be lower compared with previous studies performed in other countries, which reported a prevalence ranging from 21.7% to 79.68% [1,6,27,39,41,44,45].

For example, in Brazil, a study which focused on determining the antimicrobial susceptibility and genetic profiles of *E. coli* isolated from retail chicken meat, reported an overall prevalence of 58.66% (88 *E. coli* isolates recovered from 150 chicken meat samples) [6].

Another study recently performed in Egypt, characterizing *E. coli* isolates obtained from a variety of chicken and duck hatcheries, reported an even higher prevalence rate of 71.9% [46].

To the best of our knowledge, in Romania, at the current moment, there is little information available regarding the prevalence and AMR of *E. coli* isolated from poultry meat samples.

However, a study performed by Tabaran et al., which included only samples from intestinal content collected from cattle, reported a very high isolation rate of *E. coli* (242 isolated strains out of 250 samples) [47].

In the current study, the samples have been collected during September 2022 to June 2023, therefore also including a few cooler months, which might explain the lower-than-expected recovery of *E. coli*. It is generally considered that *E. coli* infections rates usually peak in summer [48].

When it comes to the antimicrobial susceptibility profiles of the isolated strains, all *E. coli* isolates recovered in our study proved to be susceptible to AMK, IPM, MEM, ETP and AZT. Low resistance rates to antimicrobials such as AMK, MEM or AZT have also been reported by other authors [1,12].

Most isolates proved to be resistant to TET (24/30; 80%), AMP (24/30; 80%), SMX (22/30; 73.33%), CHL (21/30; 70%) and NA (18/30; 60%). High levels of resistance to TET, as well as NA have been previously highlighted in the case of various important foodborne pathogens in our country, including *E. coli* and seems to be common and well documented [37,47]. It has been considered that such rates of resistance to these particular antimicrobials might be related to their frequent use in the prophylaxis and treatment of digestive conditions of food producing animals [49].

Moreover, resistance to AMP, as well as SMX has also been frequently reported in various countries [6,12].

Furthermore, strong resistance to CIP (17/30; 56.66%), TMP (15/30; 50%), CTX (14/30; 46.66%), CAZ (13/30; 43.33%) and GEN (12/30; 40%) was also observed. High rates of resistance to critically important antimicrobials, such as third and fourth generation cephalosporins, as well as fluoroquinolones (FQs) is concerning. High levels of resistance to FQs have also been reported by Habib et al. in a recent study performed on supermarket chicken meat samples in the United Arab Emirates (89% of the recovered isolates being resistant to CIP) [27]. On the contrary, Crecencio et al., determined a very low resistance index for the FQs class (14.96%) and a higher resistance profile for β-lactams and sulphonamides [6].

The *E. coli* isolates recovered in our study showed low percentages of resistance to FEP (7/30; 23.33%), FOX (6/30; 20%) and CST (1/30; 3.33%).

Notably, one *E. coli* isolate showed resistance to colistin, the same strain exhibiting MDR resistance to critically important antimicrobials (more than 5 different classes of antimicrobials).

Colistin, a cationic polypeptide, binds through electrostatic interactions to the phosphate group (negatively charged) of lipid A in the lipopolysaccharide of Gram-negative bacilli, disrupting its structure and ultimately causing cell death. The most frequent mechanism of acquisition of colistin resistance is related to modifications to the lipid A moiety, which lowers the affinity of colistin for lipid A and also prevents its insertion into the outer membrane. The genes that can mediate such resistance are known as the mobile colistin resistance genes (*mcr* genes) [50,51,52]. Colistin is a polymyxin antibiotic of last resort used in the treatment of infections caused by MDR Gram-negative bacteria in humans [53].

Colistin-resistant *E. coli* strains have been previously isolated in certain countries, from different sources, including chicken meat, chicken swabs and even wastewater and sludge samples [28,54,55]. For example, Kassem et al., have recently reported the isolation of a highly colistin-resistant *E. coli* from fresh chicken wings in Lebanon. The strain carried 26 antimicrobial resistance genes, among which *mcr-1.26*, a gene associated with polymyxins resistance. The authors consider the study to be the first one to report the presence of *mcr-1.26* in poultry meat worldwide [55].

To the best of our knowledge, colistin resistance in *E. coli* strains isolated from food sources has never been reported in Romania so far.

Moreover, 76.66% of the isolates proved to be multidrug resistant, showing resistance to at least three different classes of antibiotics. Resistant bacteria that reach and colonize the gastrointestinal tract via the consumption of contaminated chicken meat might represent a public health risk.

Antimicrobials have long been used in livestock as growth promoters in certain countries, as well as to prevent, control and treat different infections. The vast majority of the antimicrobial classes are used both in humans and animals, only a few classes being exclusively reserved for humans (carbapenems). Insects and some plants are also treated with antimicrobials, when necessary. Growth promoters, prophylaxis and also metaphylaxis probably account for the largest volumes of antimicrobial substances used in the food-producing animals sector [56,57]. The improper use of such antimicrobial agents in animals increases AMR to such substances, even leading to the emergence of MDR bacteria, as well as antimicrobial residues in the environment. Moreover, bacteria are also capable of transferring resistant genes to each other, thus subsequently causing and promoting multidrug resistance.

AMR resistance also has an ecological nature, being a reflection and consequence of the interconnectedness and diversity of life, including the environment, as well as human and animal health [58].

Regarding the genotypic resistance profile, the AMR determinants detected among the *E. coli* isolates were consistent with their resistance phenotypes. Most of the isolates harbored the *tetA* (16/30; 53.33%) and *tetB* (14/100; 46.66%) genes.

Tetracycline resistance is still considered a very common type of resistance in pathogenic and also commensal microorganisms. For example, in a study performed by di Francesco et al., which evaluated the prevalence of tetracycline resistance genes in broiler chickens in Tunisia, all samples were 100% positive for at least 9 of the 14 *tet* genes included in the study [59].

*blaTEM* (11/30; 36.66%), *blaCTX* (5/30; 16.66%) and *blaOXA* (5/30; 16.66%) genes have also been identified in the current study. *blaCTX* genes are common extended-spectrum- β-lactamase-producing (ESBL) types and found to be mostly associated with chicken isolates; therefore, our results are in line with data previously reported by other authors [1,42]. The common detection of *blaCTX-M* has been attributed to the worldwide off-label use of ceftiofur, a wide-spectrum antimicrobial used in veterinary medicine to treat bacterial infections in chickens [60]. Also, ESBLs can be rapidly disseminated due to the frequent horizontal gene transfer of mobile genetic elements [61].

In Romania, there is little information available regarding the isolation, identification and AMR of *E. coli* from veterinary settings. Moreover, we consider it important to mention that in our country there is no data published regarding the use of antimicrobials in broiler farms, therefore it is difficult to establish a correlation between antimicrobial use and AMR prevalence and phenotypes at farm and retail level. A recent study performed in Romania, aimed to screen and detect antibiotic residues in broiler meat based on trade system variations, seasonal differences and the impact on the safety of the consumer. The antibiotic residues which were more frequently detected belonged to the quinolones group (enrofloxacin residues being present in 84% of the samples included in the study). Other residues detected in the above-mentioned study include oxytetracycline and sulphonamides [62].

Several factors can influence the occurrence of *E. coli* in foods of animal origin, as well as the dissemination of AMR in the veterinary sector, including the improper use of antimicrobials and farm management. The lack of hygienic maintenance on farms and their surroundings, as well as the scarcity of proper knowledge among poultry farmers regarding an ideal poultry farming system are considered to play an important role in a higher prevalence of *E. coli* in poultry and poultry environments [10,38].

## 5. Conclusions

In conclusion, the study analyzed the prevalence of *E. coli* in samples of chicken meat and evaluated the antimicrobial susceptibility of the recovered isolates. Moreover, the presence of several AMR genes has also been detected.

*E. coli* was identified in 30% of the collected samples. The *E. coli* isolates recovered in the current study proved substantial resistance to multiple antibiotic classes, which is concerning.

To the best of our knowledge, this is among the first studies analyzing the prevalence and AMR of *E. coli* strains isolated from chicken meat in Romania and probably the first study reporting colistin resistance in *E. coli* isolates recovered from food sources in our country.

Among the limitations of the study, we could mention the sample size, which was relatively small, as well as the fact that the mechanism of colistin resistance has not been determined.

Furthermore, the study highlights the role of chicken meat as a reservoir of AMR *E. coli,* emphasizing the importance of continuous monitoring of the spread of AMR in the food chain.

The evaluation of the prevalence and AMR of *E. coli* is highly important, both for food safety reasons, as well as for analyzing its public health impact and the spread of AMR bacteria to humans.

## Figures and Tables

**Table 1:** Phenotypic antimicrobial susceptibility of the *E. coli* strains isolated from chicken meat.

Isolate No.	Antimicrobials	Antimicrobial Resistance Genes
1.	NA, AMP, CIP, SMX, TET	*qnrA, tetA*
2	CHL	*-*
3.	NA, AMP, CIP, SMX, TET, TMT	*qnrA, aadA1, tetA*
4.	NA, AMP, CTX, CAZ, CIP, CHL, GEN, TET	*blaTEM, tetA, tetB*
5.	CTX, CAZ	*-*
6.	NA, AMP, CIP, CHL, GEN, TET	*aac, tetB*
7.	CTX, CAZ	*blaCTX*
8.	NA, AMP, CTX, CAZ, CIP, CHL, SMX, TET, TMT	*blaTEM, blaOXA, tetA*
9.	NA, AMP, CTX, CAZ, CIP, CHL, GEN, SMX, TET, TMT	*blaTEM, blaCTX, aadA1, sul1, tetA*
10.	NA, AMP, CTX, CAZ, CIP, CHL, GEN, SMX, TET, TMT	*blaTEM, blaOXA, aadA1, sul1, tetA*
11.	NA, AMP, CTX, CAZ, CIP, CHL, GEN, SMX, TET, TMT	*blaTEM, blaOXA, sul1*
12.	NA, AMP, CIP, CHL, SMX, TET, TMT	*qnrA, aadA1, tetA, tetB*
13.	AMP, CHL, SMX, TET, TMT	*aadA1, tetB*
14.	NA, AMP, CIP, CHL, SMX, TET, TMT	*aadA1, sul1*
15.	AMP, SMX, TET, TMT	*tetA, tetB*
16.	NA, AMP, CIP, CHL, GEN, SMX, TET	*blaTEM, aac, sul1*
17.	AMP, CHL, SMX, TET	*sul1, tetA*
18.	SMX, TET, TMT	*tetA, tetB*
19.	SMX	*sul1*
20.	NA, AMP, CIP, TET	*qnrA, tetB*
21.	NA, AMP, CIP, CHL, GEN, SMX, TET	*qnrA, aac, sul1, tetA, tetB*
22.	-	*-*
23.	AMP, CHL, SMX, TET, TMT	*aadA1, tetA, tetB*
24.	NA, AMP, CTX, CAZ, CIP, CHL, CST, GEN, SMX, TET, FEP	*blaTEM, blaCTX, tetA, tetB*
25.	NA, AMP, CTX, CIP, CHL, GEN, SMX, TET, TMT, FEP, FOX	*blaTEM, blaOXA, tetA, tetB*
26.	NA, AMP, CTX, CAZ, CIP, CAZ, CHL, GEN, SMX, TET, TMT, FEP, FOX	*blaTEM, blaOXA, blaCTX, tetA*
27.	NA, AMP, CTX, CAZ, CIP, CHL, SMX, TET, FEP, FOX	*blaTEM, tetB*
28.	NA, AMP, CTX, CAZ, CHL, GEN, SMX, TET, FEP, FOX	*blaTEM, blaCTX, tetB*
29.	AMP, CTX, CAZ, CHL, GEN, SMX, TET, TMT, FEP, FOX	*tetA*
30.	AMP, CTX, CAZ, CHL, SMX, TET, FEP, FOX	*tetB*

NA: nalidixic acid, GEN: gentamicin, AMP: ampicillin, CTX: cefotaxime, CAZ: ceftazidime, FEP: cefepime, FOX: cefoxitin, CIP: ciprofloxacin, CHL: chloramphenicol, CST: colistin, TET: tetracycline, TMT: trimethoprim, SMX: sulfamethoxazole.

## Data Availability

Data are contained within the article.

## References

[1] Xedzro C., Kimura T., Shimamoto T., Ahmed A.M., Shimamoto T. (2023). Comparative molecular profiling of antimicrobial resistance and phylogenetic characterization of multidrug-resistant *Escherichia coli* isolated from meat sources in 2009 and 2021 in Japan. Int. J. Food Microbiol..

[2] Taggar G., Rehman M.A., Yin X., Lepp D., Ziebell K., Handyside P., Boerlin P., Diarra M.S. (2018). Antimicrobial-Resistant *E. coli* from Surface Waters in Southwest Ontario Dairy Farms. J. Environ. Qual..

[3] Bendary M.M., Abdel-Hamid M.I., Alshareef W.A., Alshareef H.M., Mosbah R.A., Omar N.N., Al-Sanea M.M., Alhomrani M., Alamri A.S., Moustafa W.H. (2022). Comparative Analysis of Human and Animal *E. coli*: Serotyping, Antimicrobial Resistance, and Virulence Gene Profiling. Antibiotics.

[4] Ramos S., Silva V., de Lurdes Enes Dapkevicius M., Caniça M., Tejedor-Junco M.T., Igrejas G., Poeta P. (2020). *Escherichia coli* as Commensal and Pathogenic Bacteria among Food-Producing Animals: Health Implications of Extended Spectrum β-Lactamase (ESBL) Production. Animals.

[5] Pereira A., Santos A., Tacão M., Alves A., Henriques I., Correia A. (2013). Genetic diversity and antimicrobial resistance of *Escherichia coli* from Tagus estuary (Portugal). Sci. Total Environ..

[6] Crecencio R.B., Brisola M.C., Bitner D., Frigo A., Rampazzo L., Borges K.A., Furian T.Q., Salle C.T.P., Moraes H.L.S., Faria G.A. (2020). Antimicrobial susceptibility, biofilm formation and genetic profiles of *Escherichia coli* isolated from retail chicken meat. Infect. Genet. Evol..

[7] Ghosh S., Bornman C., Zafer M.M. (2021). Antimicrobial Resistance Threats in the emerging COVID-19 pandemic: Where do we stand?. J. Infect. Public Health.

[8] Rawat N., Yadav K., Jamwal R., Sabu B., Bandyopadhyay A., Rajagopal R. (2023). Prevalence of multiple-drug resistance and identification of underlying antibiotic resistance genes in *Escherichia coli* isolated from ready-to-eat chicken salads being sold in restaurants in Delhi, India. Food Humanit..

[9] World Health Organisation (WHO) Antimicrobial Resistance. https://www.who.int/news-room/fact-sheets/detail/antimicrobial-resistance.

[10] Islam M.S., Hossain M.J., Sobur M.A., Punom S.A., Rahman A.M.M.T., Rahman M.T. (2023). A Systematic Review on the Occurrence of Antimicrobial-Resistant *Escherichia coli* in Poultry and Poultry Environments in Bangladesh between 2010 and 2021. BioMed Res. Int..

[11] Islam M.S., Sobur M.A., Rahman S., Ballah F.M., Ievy S., Siddique M.P., Rahman M., Kafi M.A., Rahman M.T. (2022). Detection of blaTEM, blaCTX-M, blaCMY, and blaSHV Genes Among Extended-Spectrum Beta-Lactamase-Producing *Escherichia coli* Isolated from Migratory Birds Travelling to Bangladesh. Microb. Ecol..

[12] Yang C., Rehman M.A., Yin X., Carrillo C.D., Wang Q., Yang C., Gong J., Diarra M.S. (2021). Antimicrobial resistance phenotypes and genotypes of *Escherichia coli* isolates from broiler chickens fed encapsulated cinnamaldehyde and citral. J. Food Prot..

[13] Talukder M., Islam M.S., Ievy S., Sobur M.A., Ballah F.M., Najibullah M., Rahman M.B., Rahman M.T., Khan M.F.R. (2021). Detection of multidrug resistant *Salmonella* spp. from healthy and diseased broilers having potential public health significance. J. Adv. Biotechnol. Exp. Ther..

[14] Hussain H.I., Aqib A.I., Seleem M.N., Shabbir M.A., Hao H., Iqbal Z., Kulyar M.F.e.A., Zaheer T., Li K. (2021). Genetic basis of molecular mechanisms in β-lactam resistant gram-negative bacteria. Microb. Pathog..

[15] Dunn G., Bakker K., Harris L. (2014). Drinking Water Quality Guidelines across Canadian provinces and territories: Jurisdictional variation in the context of decentralized water governance. Int. J. Environ. Res. Public Health.

[16] Benameur Q., Tali-Maamar H., Assaous F., Guettou B., Rahal K., Ben-Mahdi M.H. (2019). Detection of multidrug resistant *Escherichia coli* in the ovaries of healthy broiler breeders with emphasis on extended-spectrum β-lactamases producers. Comp. Immunol. Microbiol. Infect. Dis..

[17] Nyirabahizi E., Tyson G.H., Dessai U., Zhao S., Kabera C., Crarey E., Womack N., Crews M.K., Strain E., Tate H. (2020). Evaluation of *Escherichia coli* as an indicator for antimicrobial resistance in Salmonella recovered from the same food or animal ceca samples. Food Control.

[18] Ouchar Mahamat O., Kempf M., Lounnas M., Tidjani A., Hide M., Benavides J.A., Carrière C., Bañuls A.L., Jean-Pierre H., Ouedraogo A.S. (2021). Epidemiology and prevalence of extended-spectrum β-lactamase- and carbapenemase-producing Enterobacteriaceae in humans, animals and the environment in West and Central Africa. Int. J. Antimicrob. Agents.

[19] Partridge S.R., Kwong S.M., Firth N., Jensen S.O. (2018). Mobile genetic elements associated with antimicrobial resistance. Clin. Microbiol. Rev..

[20] Razazi K., Derde L.P.G., Verachten M., Legrand P., Lesprit P., Brun-Buisson C. (2012). Clinical impact and risk factors for colonization with extended-spectrum β-lactamase-producing bacteria in the intensive care unit. Intensive Care Med..

[21] Xiao L., Wang X., Kong N., Zhang L., Cao M., Sun M., Wei Q., Liu W. (2019). Characterization of Beta-Lactamases in Bloodstream-Infection *Escherichia coli*: Dissemination of bla_ADC–162_ and bla_CMY–2_ Among Bacteria via an IncF Plasmid. Front. Microbiol..

[22] Colobatiu L., Tabaran A., Flonta M., Oniga O., Mirel S., Mihaiu M. (2015). First description of plasmid-mediated quinolone resistance determinants and β-lactamase encoding genes in non-typhoidal Salmonella isolated from humans, one companion animal and food in Romania. Gut Pathog..

[23] Nguyen D.P., Nguyen T.A.D., Le T.H., Tran N.M.D., Ngo T.P., Dang V.C., Kawai T., Kanki M., Kawahara R., Jinnai M. (2016). Dissemination of Extended-Spectrum β-Lactamase- and AmpC β-Lactamase-Producing *Escherichia coli* within the Food Distribution System of Ho Chi Minh City, Vietnam. BioMed Res. Int..

[24] Seo K.W., Lee Y.J. (2019). Characterization of plasmid mediated quinolone resistance determinants in ciprofloxacin resistant-*Escherichia coli* from chicken meat produced by integrated broiler operations in Korea. Int. J. Food Microbiol..

[25] Wasyl D., Hoszowski A., Zaja̧c M., Szulowski K. (2013). Antimicrobial resistance in commensal *Escherichia coli* isolated from animals at slaughter. Front. Microbiol..

[26] Zainab L., Ibrar K., Sadiq A., Hamid A.K., Ullah M., Noor R. (2022). Extended spectrum beta lactamases-producing *Escherichia coli* in retail chicken meat from Khyber Pakhtunkhwa, Pakistan. Saudi J. Biol. Sci..

[27] Habib I., Elbediwi M., Mohamed M.Y.I., Ghazawi A., Abdalla A., Khalifa H.O., Khan M. (2023). Enumeration, antimicrobial resistance and genomic characterization of extended-spectrum β-lactamases producing *Escherichia coli* from supermarket chicken meat in the United Arab Emirates. Int. J. Food Microbiol..

[28] Zhang W., Zhang T., Wang C., Liang G., Lu Q., Wen G., Guo Y., Cheng Y., Wang Z., Shao H. (2022). Prevalence of colistin resistance gene mcr-1 in *Escherichia coli* isolated from chickens in central China, 2014 to 2019. J. Glob. Antimicrob. Resist..

[29] Dadashi M., Sameni F., Bostanshirin N., Yaslianifard S., Khosravi-Dehaghi N., Nasiri M.J., Goudarzi M., Hashemi A., Hajikhani B. (2022). Global prevalence and molecular epidemiology of mcr-mediated colistin resistance in *Escherichia coli* clinical isolates: A systematic review. J. Glob. Antimicrob. Resist..

[30] Kaye K.S., Pogue J.M., Tran T.B., Nation R.L., Li J. (2016). Agents of Last Resort: Polymyxin Resistance. Infect. Dis. Clin. N. Am..

[31] Tabaran A., Mihaiu M., Tăbăran F., Colobatiu L., Reget O., Borzan M.M., Dan S.D. (2016). First study on characterization of virulence and antibiotic resistance genes in verotoxigenic and enterotoxigenic *E. coli* isolated from raw milk and unpasteurized traditional cheeses in Romania. Folia Microbiol..

[32] Beres C., Colobatiu L., Tabaran A., Mihaiu R., Iuhas C., Mihaiu M. (2022). Clostridioides difficile in Food-Producing Animals in Romania: First Study on the Prevalence and Antimicrobial Resistance. Antibiotics.

[33] Beres C., Colobatiu L., Tabaran A., Mihaiu R., Mihaiu M. (2023). Prevalence and Characterisation of Clostridium perfringens Isolates in Food-Producing Animals in Romania. Microorganisms.

[34] (2001). Microbiology-Horizontal Method for the Detection of Escherichia coli O157.

[35] CLSI (2018). Performance standards for antimicrobial susceptibility testing. 28th Edition Informational Supplement. M100.

[36] Mihaiu L., Lapusan A., Tanasuica R., Sobolu R., Mihaiu R., Oniga O., Mihaiu M. (2014). First study of Salmonella in meat in Romania. J. Infect. Dev. Ctries..

[37] Chirila F., Tabaran A., Fit N., Nadas G., Mihaiu M., Tabaran F., Cătoi C., Reget O.L., Dan S.D. (2017). Concerning Increase in Antimicrobial Resistance in Shiga Toxin-Producing *Escherichia coli* Isolated from Young Animals during 1980–2016. Microbes Environ..

[38] Telli A.E., Biçer Y., Telli N., Güngör C., Turkal G., Onmaz N.E. (2022). Pathogenic *Escherichia coli* and *Salmonella* spp. in Chicken Carcass Rinses: Isolation and Genotyping by ERIC-PCR. Pak. Vet. J..

[39] Garcia-Graells C., Berbers B., Verhaegen B., Vanneste K., Marchal K., Roosens N.H.C., Botteldoorn N., De Keersmaecker S.C.J. (2020). First detection of a plasmid located carbapenem resistant blaVIM-1 gene in *E. coli* isolated from meat products at retail in Belgium in 2015. Int. J. Food Microbiol..

[40] Díaz-Jiménez D., García-Meniño I., Fernández J., García V., Mora A. (2020). Chicken and turkey meat: Consumer exposure to multidrug-resistant Enterobacteriaceae including mcr-carriers, uropathogenic *E. coli* and high-risk lineages such as ST131. Int. J. Food Microbiol..

[41] Barilli E., Vismarra A., Frascolla V., Rega M., Bacci C. (2020). *Escherichia coli* Strains Isolated from Retail Meat Products: Evaluation of Biofilm Formation Ability, Antibiotic Resistance, and Phylogenetic Group Analysis. J. Food Prot..

[42] Eltai N.O., Yassine H.M., El-Obeid T., Al-Hadidi S.H., Al Thani A.A., Alali W.Q. (2020). Prevalence of antibiotic-resistant *Escherichia coli* isolates from local and imported retail chicken carcasses. J. Food Prot..

[43] Wang M., Jiang M., Wang Z., Chen R., Zhuge X., Dai J. (2021). Characterization of antimicrobial resistance in chicken-source phylogroup F *Escherichia coli*: Similar populations and resistance spectrums between *E. coli* recovered from chicken colibacillosis tissues and retail raw meats in Eastern China. Poult. Sci..

[44] Patel S., Srivastava S., Rawat M., Singh D. (2018). International Journal of Biological Macromolecules Preparation and optimization of chitosan-gelatin films for sustained delivery of lupeol for wound healing. Int. J. Biol. Macromol..

[45] Vincent C., Boerlin P., Daignault D., Dozois C.M., Dutil L., Galanakis C., Reid-Smith R.J., Tellier P.P., Tellis P.A., Ziebell K. (2010). Food reservoir for *Escherichia coli* causing urinary tract infections. Emerg. Infect. Dis..

[46] Yousef H.M.Y., Hashad M.E., Osman K.M., Alatfeehy N.M., Hassan W.M.M., Elebeedy L.A., Salem H.M., Shami A., Al-Saeed F.A., El-Saadony M.T. (2023). Surveillance of *Escherichia coli* in different types of chicken and duck hatcheries: One health outlook. Poult. Sci..

[47] Tabaran A., Soulageon V., Chirila F., Reget O.L., Mihaiu M., Borzan M., Dan S.D. (2022). Pathogenic *E. coli* from Cattle as a Reservoir of Resistance Genes to Various Groups of Antibiotics. Antibiotics.

[48] Deeny S.R., van Kleef E., Bou-Antoun S., Hope R.J., Robotham J.V. (2015). Seasonal changes in the incidence of *Escherichia coli* bloodstream infection: Variation with region and place of onset. Clin. Microbiol. Infect..

[49] Economou V., Gousia P. (2015). Agriculture and food animals as a source of antimicrobial-resistant bacteria. Infect. Drug Resist..

[50] Deris Z.Z., Akter J., Sivanesan S., Roberts K.D., Thompson P.E., Nation R.L., Li J., Velkov T. (2013). A secondary mode of action of polymyxins against Gram-negative bacteria involves the inhibition of NADH-quinone oxidoreductase activity. J. Antibiot..

[51] Li Z., Velkov T. (2019). Polymyxins: Mode of Action. Adv. Exp. Med. Biol..

[52] Ling Z., Yin W., Shen Z., Wang Y., Shen J., Walsh T.R. (2020). Epidemiology of mobile colistin resistance genes mcr-1 to mcr-9. J. Antimicrob. Chemother..

[53] Humphrey M., Larrouy-Maumus G.J., Furniss R.C.D., Mavridou D.A.I., Sabnis A., Edwards A.M. (2021). Colistin resistance in *Escherichia coli* confers protection of the cytoplasmic but not outer membrane from the polymyxin antibiotic. Microbiology.

[54] Wang D., Zou H., Zhao L., Li Q., Meng M., Li X., Berglund B. (2023). High prevalence of *Escherichia coli* co-harboring conjugative plasmids with colistin- and carbapenem resistance genes in a wastewater treatment plant in China. Int. J. Hyg. Environ. Health.

[55] Kassem I.I., Osman M., Hassan J., Sulaiman A.A., Mann D., Esseili M.A., Naas T., Deng X. (2023). First report of the mobile colistin resistance gene, mcr-1.26, in multidrug-resistant *Escherichia coli* isolated from retail chicken meat. J. Glob. Antimicrob. Resist..

[56] McEwen S.A., Fedorka-Cray P.J. (2002). Antimicrobial use and resistance in animals. Clin. Infect. Dis..

[57] Collignon P.J., McEwen S.A. (2019). One Health—Its Importance in Helping to Better Control Antimicrobial Resistance. Trop. Med. Infect. Dis..

[58] Robinson T.P., Bu D.P., Carrique-Mas J., Fèvre E.M., Gilbert M., Grace D., Hay S.I., Jiwakanon J., Kakkar M., Kariuki S. (2016). Antibiotic resistance is the quintessential One Health issue. Trans. R. Soc. Trop. Med. Hyg..

[59] Di Francesco A., Salvatore D., Sakhria S., Catelli E., Lupini C., Abbassi M.S., Bessoussa G., Ben Yahia S., Ben Chehida N. (2021). High Frequency and Diversity of Tetracycline Resistance Genes in the Microbiota of Broiler Chickens in Tunisia. Animals.

[60] Casella T., Nogueira M.C.L., Saras E., Haenni M., Madec J.Y. (2017). High prevalence of ESBLs in retail chicken meat despite reduced use of antimicrobials in chicken production, France. Int. J. Food Microbiol..

[61] Verraes C., Van Boxstael S., Van Meervenne E., Van Coillie E., Butaye P., Catry B., de Schaetzen M.-A., Van Huffel X., Imberechts H., Dierick K. (2013). Antimicrobial resistance in the food chain: A review. Int. J. Environ. Res. Public Health.

[62] Pogurschi E.N., Grigore D.-M., Ianitchi D., Bahaciu G., Popa D.C., Dragomir N., Pet I. (2023). Screening and detection of antibiotic residues on broiler meat based on trade system variations, seasonal differences, and the impact on final consumer safety in Romania. Front. Sustain. Food Syst..

